# Performance Evaluation of Rubber Modified Asphalt Mixtures with Two Typical Light Oils: A Comparative Study Between Aromatic and Tall Oils

**DOI:** 10.3390/ma19030508

**Published:** 2026-01-27

**Authors:** Qiangbin Zhu, Youxin Jiang, Dongdong Ge, Li Liu, Chaopeng Li, Xiangyang Jiang, Milkos Borges Cabrera

**Affiliations:** 1 Gansu Province Transportation Planning Survey & Design Institute Co., Ltd., Lanzhou 730030, China; jx13739318068@163.com (Q.Z.); jyx3016@163.com (Y.J.); 2 National Key Laboratory of Green and Long-Life Road Engineering in Extreme Environment (Changsha), Changsha University of Science & Technology, Changsha 410114, China; liuli@csust.edu.cn (L.L.); milkos_csust@126.com (M.B.C.); 3 National Engineering Research Center of Highway Maintenance Technology, Changsha University of Science & Technology, Changsha 410114, China; 4 Gansu He-Sai Highway Construction and Development Co., Ltd., Hezuo 747200, China; lichaopeng0617@163.com

**Keywords:** rubber modified asphalt, light oil, performance evaluation, high-temperature performance, low-temperature cracking

## Abstract

Recycling waste rubber is essential for promoting circular economy practices, reducing environmental pollution, and conserving resources. This study examines the performance of crumb rubber-modified asphalt mixtures incorporating two light oils (aromatic oil and tall oil) to alleviate the high viscosity and poor workability of asphalt with high rubber content. Mixtures were prepared using a neat asphalt modified with 20% crumb rubber and 5% light oil (by mass of the neat asphalt), combined with basalt aggregate in an AC-13 gradation. High-temperature performance was evaluated via Marshall stability and wheel tracking tests at 60 °C, moisture sensitivity through immersion Marshall and freeze–thaw splitting tests, and low-temperature cracking resistance using semi-circular bending (SCB) tests at 15 °C. Tensile strength and fatigue life were measured by splitting tests at 25 °C and fatigue tests at 15 °C, respectively. Results indicate that the rubber-modified mixtures showed significant improvements: the total deformation decreased by 44.7% and 64.1% for aromatic oil- and tall oil-modified mixtures, respectively, compared to the neat asphalt. Fracture toughness increased by 46.5% and 71.9%, and tensile strength improved by 40.2% and 63.6%, respectively. At a low stress ratio (0.281), mixtures with tall oil exhibited a 47.9% longer fatigue life than those with aromatic oil. Tall oil demonstrated superior performance, attributed to enhanced rubber swelling and crosslinked network formation, which improved viscosity and aggregate coating. The findings confirm that light oil-modified rubber asphalt mixtures, especially those containing tall oil, present a viable approach for developing high-performance and environmentally sustainable road pavements.

## 1. Introduction

The growing demand for transportation infrastructure continues to drive the consumption of rubber resources. While rubber production increases annually, its recovery rate remains relatively low [1,2]. Consequently, the recycling and management of scrap rubber have become pressing issues. The environmental challenges posed by accumulating scrap rubber cannot be overlooked [3]. Common disposal methods include material recycling and energy recovery [4]; lifecycle assessments suggest that material recycling generally has a lower environmental impact [5].

In pavement engineering, crumb rubber from waste tires is utilized as an asphalt modifier [6]. While asphalt mixture production is linked to greenhouse gas emissions and energy consumption, incorporating crumb rubber can mitigate heat loss during construction, thereby conserving energy [7]. Moreover, pavements constructed with rubber-modified asphalt mixtures typically result in lower greenhouse gas emissions [8]. Additionally, such mixtures help reduce road traffic noise. For example, Margorínová et al. reported that using rubber granulate in stone mastic asphalt (SMA) reduced traffic noise levels by 3.2 dB (2011) and 3.3 dB (2017) compared to conventional asphalt layers, as measured by the Statistical Pass-By Index (SPBI) according to EN ISO 11819-1 [9]. These multifaceted environmental benefits further support the use of rubber-modified asphalt in sustainable pavement applications.

Currently, the preparation of rubber-modified asphalt primarily follows two distinct procedures: the dry process and the wet process. In the dry process, crumb rubber is first mixed with aggregates, after which asphalt is added to produce the rubber-modified asphalt mixture [10]. Conversely, the wet process involves the pre-blending of crumb rubber with asphalt to create a homogeneous rubber-modified asphalt binder, which is subsequently mixed with aggregates [2].

In the dry process, crumb rubber can partially replace fine aggregates, significantly enhancing the mixture’s crack resistance [10]. However, the performance of mixtures with high rubber content tends to deteriorate, with an optimal content identified around 20% [11]. Achieving adequate field compaction with the dry process also presents challenges, potentially compromising the moisture resistance of the pavement [12]. In response to these limitations, research has proposed several improvements. Using finer, mechanically processed crumb rubber can yield modified asphalt mixtures with superior performance [13]. Furthermore, microwave activation of the rubber [14] or its pre-treatment with wax additives [15] can improve the mechanical properties of the resulting mixtures.

Rubber-modified asphalt mixtures produced via the wet process exhibit improved moisture stability and mechanical performance [16]. However, incorporating a high content of crumb rubber (CR) increases the viscosity of the asphalt binder. A larger crumb rubber particle size produces a similar effect [17], which can lead to practical difficulties during construction [18]. Optimizing the preparation conditions for rubber-modified asphalt allows for the regulation of the crumb rubber swelling process, facilitating the production of a superior modified binder [2]. Furthermore, the composite modification of neat asphalt with crumb rubber alongside polymers such as polyethylene (PE), resin, or styrene–butadiene–styrene copolymer (SBS) can yield asphalt mixtures with enhanced performance [19].

Light oil generally refers to hydrocarbon mixtures, which in the petroleum refining industry denotes light fraction oils or light oil products [20]. Common light oils employed in asphalt modification include paraffinic, aromatic, fatty acid, and turpentine oils. Among these, fatty acids derived from bio-oils are most frequently utilized in modified asphalt [21]. The use of waste cooking oil to activate crumb rubber can enhance the storage stability of the prepared modified asphalt binder [22]. Incorporating bio-oils into modified asphalt can improve its high-temperature performance and enhance the workability of the mixture [23]. The average molecular weight between crosslinks in an aromatic oil (AO) blend is reported to be 40.3% higher than that in a paraffinic oil (PO) blend, while the PO blend exhibits a lower Mooney viscosity than the AO/isobutylene–isoprene rubber (IIR) blend [24]. The temperature sensitivity of tall oil and boron oxide composite-modified asphalt is reduced [25]. The absorption and release of light components by crumb rubber (CR) can improve the aging resistance of asphalt [26]. Mineral oil, particularly aromatic oil, can enhance the compatibility in natural rubber-modified asphalt (NRMA) [27]. Light oils can replenish the light components in asphalt systems and promote the dispersion of asphaltene, resin, and rubber [28]. Incorporating 2% to 4% bio-oil into asphalt mixtures does not impair their rutting or low-temperature performance [29].

Although crumb rubber modification can enhance asphalt performance, the inherently high viscosity of rubber-modified asphalt necessitates elevated mixing temperatures, leading to significantly increased energy consumption [2]. Light oils can improve the workability of rubber-modified asphalt by reducing its viscosity [23]. Existing research has primarily focused on the rheological properties and modification mechanisms of light oil-modified rubber asphalt binders. However, systematic investigations at the mixture performance level remain relatively scarce, and there is limited assessment of how light oil selection influences the overall performance of asphalt mixtures under various service conditions. This knowledge gap hinders the broader application of light oil-modified rubber asphalt.

Therefore, this study developed a preparation method for light oil/rubber composite-modified asphalt aimed at reducing production temperatures while meeting performance specifications. The effects of two distinct light oils (aromatic oil and tall oil) on the pavement performance and mechanical characteristics of rubber-modified asphalt mixtures were compared. Experimental results elucidate the enhancement mechanism and effectiveness of light oil-modified rubber asphalt mixtures, leading to the identification of a more suitable light oil type. This work provides practical guidance for optimizing material selection based on engineering requirements, bridges the gap between laboratory binder testing and actual pavement application, and contributes to the development of more durable and sustainable rubber-modified asphalt pavements.

## 2. Materials and Preparation Procedures

### 2.1. Materials

The materials used in this study included aromatic oil, tall oil, 70# asphalt, basalt aggregate, and an antistripping agent. The fundamental properties of the 70# neat asphalt are presented in Table 1.

The aromatic oil and tall oil used in this study were supplied by Nantong Yuhao Chemical Technology Co., Ltd., Nantong, China. Their key performance indicators are listed in Table 2 and Table 3. These two light oils were selected for their distinct characteristics and relevance to sustainability. Tall oil is a by-product of the wood pulping kraft process, consisting of approximately 69.33% fatty acids, and serves as a representative renewable bio-oil [35]. In contrast, aromatic oil is a petroleum distillate with about 95% aromatic content, representing a conventional petroleum-based modifier [36]. This selection allows for a direct comparison between bio-based and petroleum-based alternatives, addressing both performance and environmental sustainability objectives [37,38].

**Figure 1:**
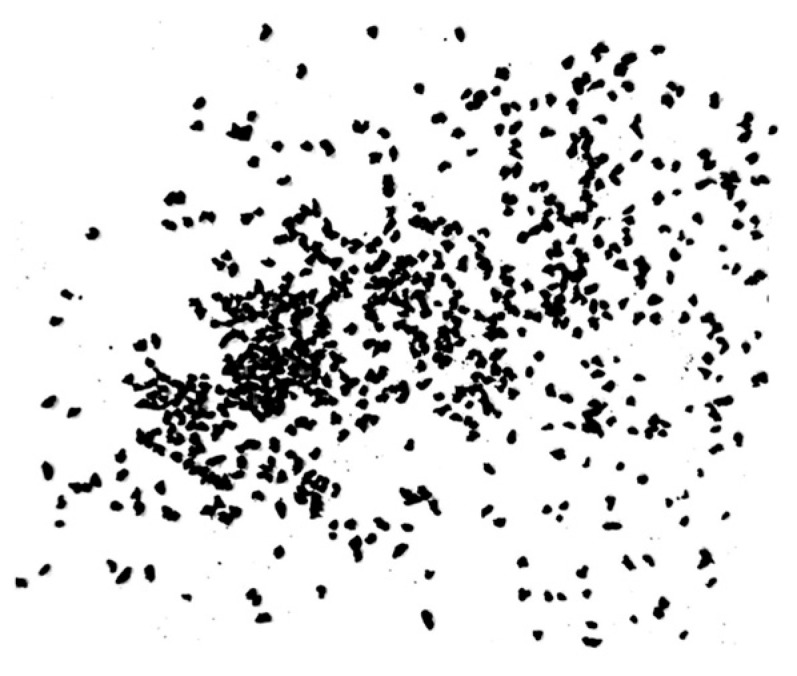
Crumb rubber.

The crumb rubber was provided by Guangxi Transportation Science and Technology Group Co., Ltd., Nanning, China. The material was produced by crushing waste tires and had a particle size of 50 mesh. The fundamental physical parameters of the crumb rubber are presented in Table 4. The crumb rubber is shown in Figure 1.

Basalt was used as the aggregate in the performance testing of the asphalt mixtures. The aggregate’s relevant properties are presented in Table 5. The test results for the mineral powder filler used in this study are shown in Table 6.

**Figure 2:**
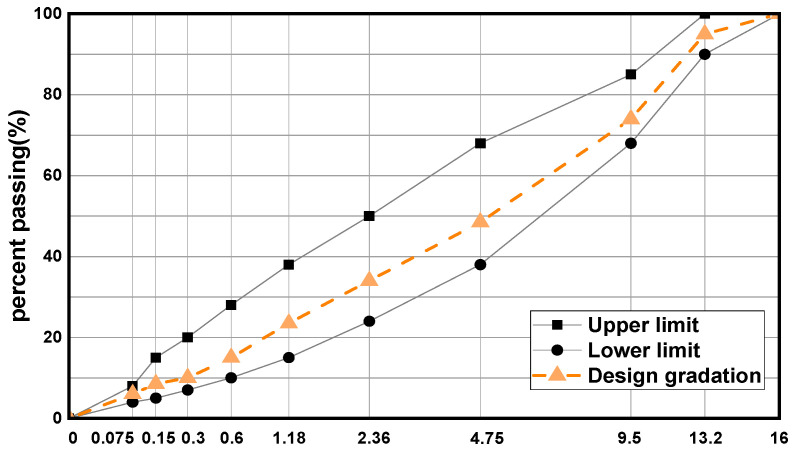
Grading curve chart.

The gradation design of the asphalt mixture followed the widely adopted AC-13 type. The corresponding gradation curve is illustrated in Figure 2.

### 2.2. Preparation Procedure for Test Specimens

The crumb rubber and light oil were incorporated into the neat asphalt using an external blending method, with their dosages expressed as percentages by mass of the neat asphalt. The preparation procedure was as follows. First, 500 g of the neat asphalt was heated in an oven at 135 °C for 1 h until it became fluid. Subsequently, 5% (25 g) of the specified light oil was gradually added to the neat asphalt and manually stirred with a glass rod for initial mixing. The blend was then sheared at 135 °C and 3500 rpm for 5 min using a high-speed shear mixer to obtain a homogeneous light oil–asphalt binder. This binder was heated to 160 °C. Then, 20% (100 g) of crumb rubber was added incrementally with manual stirring to prevent agglomeration. Finally, the mixture was sheared at 160 °C and 3500 rpm for 30 min to produce the final light oil/rubber composite-modified asphalt. This preparation method aligns with that described in a previous study [21].

The mixtures were prepared using the two different oils according to the following procedure. The mixer was preheated at 170 °C for 4 h before the aggregate was placed into it. Simultaneously, the prepared modified asphalt was stored in an oven at 170 °C to minimize heat loss. Once the target temperature was reached, the modified asphalt was transferred to the mixing tank and mixed with the aggregate for 90 s to ensure homogeneity. Subsequently, the mixture was cured in an oven at 170 °C for 0.5 h. This curing step promotes uniform binder coating, further swelling of the crumb rubber, and development of its network structure, thereby enhancing the mixture’s bond strength and durability. This preparation protocol, consistent with a previous study [39], aims to improve the stability of mixture performance under controlled laboratory conditions. After curing, the mixture was immediately compacted into specimens. For the control group, specimens of the neat asphalt mixture were prepared at 160 °C without undergoing the curing process. The types of asphalt mixtures investigated in this study are detailed in Table 7.

### 2.3. Optimum Asphalt–Aggregate Ratio Determination Procedure

The Marshall test method was employed to determine the optimum asphalt content for each mixture, targeting an air void content of 4% [40]. The test results are presented in Table 8. The optimum asphalt–aggregate ratio was determined as 4.38% for the neat asphalt mixture, 6.0% for the AO, and 5.9% for the TO. When the masses of the light oil and crumb rubber were deducted, the effective base asphalt content was 3.29% for the neat asphalt mixture, 4.5% for the AO, and 4.43% for the TO.

## 3. Performance Experimental Procedures

### 3.1. Rheological Properties

The rheological properties of the asphalt binders were evaluated using a Dynamic Shear Rheometer (DSR, Anton Paar, Graz, Austria) and a Low-Temperature Bending Beam Rheometer (BBR, Cannon Instrument Company, State College, PA, USA) over a specified temperature range. BBR tests were conducted to assess the low-temperature cracking resistance at −18 °C and −12 °C, following the AASHTO T 313-19 standard [41]. A constant test load of 980 ± 50 mN was applied. The creep stiffness (S-value) was calculated using Equation (1).

(1) $$S(t)=\frac{PL^{3}}{4bh^{3}\delta (t)}$$

where *S*(*t*) is the flexural creep stiffness at time *t*, in MPa; *P* is the applied load, in N; *L* is the span length, in mm; *b* and *h* are the width and height of the specimen, in mm; and *δ*(*t*) is the mid-span deflection of the specimen at time *t*, in mm. The m-value, which is the slope of the logarithm of creep stiffness versus the logarithm of time, is calculated using Equation (2).

(2) $$m(t)=\left|\frac{d\{\log[S(t)]\}}{d[\log(t)]}\right|$$

Temperature sweep (TS) tests were performed on unaged and RTFO-aged asphalt binders using a dynamic shear rheometer (Anton Paar MCR 702e, Anton Paar, Graz, Austria) in accordance with AASHTO T 315-20 [42]. The tests were conducted over a temperature range of 70 to 76 °C at a fixed frequency of 10 rad/s.

### 3.2. Water Stability

The water stability of the rubber-modified asphalt mixtures was evaluated using the immersion Marshall test and the freeze–thaw splitting test.

#### 3.2.1. Immersion Marshall Test

The immersion Marshall test was conducted according to the standard [43] using two sets of specimens. The stability (MS) of the first set was determined after soaking the specimens in a water bath at 60 °C for 2 h. The second set of specimens was immersed in a water bath at 60 °C for 48 h, and their stability (MS_1_) was then measured. All Marshall tests were performed at a loading rate of 50 mm/min.

#### 3.2.2. Freeze–Thaw Splitting Test

The freeze–thaw splitting test was conducted in accordance with the AASHTO T283-22 standard [44]. Two sets of Marshall specimens were prepared: one subjected to freeze–thaw conditioning and the other serving as the unconditioned control. The splitting tensile strengths of both specimen sets (RT1 and RT2, respectively) were measured independently. The freeze–thaw splitting strength ratio was then calculated. All tests were performed at 25 °C using a loading rate of 50 mm/min [45].

### 3.3. High-Temperature Stability

The high-temperature stability of the asphalt mixtures was evaluated using the Marshall test and the rutting test.

#### 3.3.1. Marshall Test

The Marshall specimens were maintained at 60 °C in a water bath. The Marshall stability test was then performed at a loading rate of 50 mm/min. Each mixture group consisted of four parallel specimens for the determination of stability and flow values.

#### 3.3.2. Rutting Test

Three parallel rut specimens measuring 300 mm × 300 mm × 50 mm were prepared. Prior to testing, the specimens were conditioned in an incubator at 60 °C for 5 h. The wheel tracking test was conducted with a wheel pressure of 0.7 MPa. The dynamic stability (DS) was calculated from the deformation data recorded between 45 and 60 min after the test commenced [40].

Total deformation and dynamic stability were used as indicators to evaluate the high-temperature stability of the asphalt mixtures. The total deformation was defined as the permanent vertical deformation (in mm) of the specimen surface at the end of the test. The formula for calculating dynamic stability is presented in Equation (3).

(3) $$DS=\frac{(t_{2}-t_{1})\times N\times C_{1}\times C_{2}}{d_{2}-d_{1}}$$

where *DS* is the dynamic stability from the wheel tracking test (in cycles/mm); *C*_1_ is the testing machine type coefficient, taken as 1; *C*_2_ is the specimen coefficient, taken as 1; *t*_1_ is 45 min; *t*_2_ is 60 min; *d*_1_ and *d*_2_ are the rut depths (in mm) at times *t*_1_ and *t*_2_, respectively; and *N* is the loading speed, taken as 42 passes per minute.

### 3.4. Low-Temperature Cracking Test

The low-temperature crack resistance of the asphalt mixtures was assessed using the Semi-Circular Bending (SCB) test [46]. The test, based on linear elastic fracture mechanics, assesses the material’s resistance to crack propagation at low temperatures. SCB specimens were prepared by cutting a semi-circular geometry from a Marshall compacted specimen with a diameter of 150 mm. The final SCB specimen had a thickness of 24.7 mm, a height of 115 mm, and a 1.5 mm wide notch with a depth of 15 mm introduced at the center of the straight edge. The test was conducted at 15 °C. Representative load–displacement curves, along with the detailed equations for calculating fracture energy and fracture toughness, are provided in the AASHTO TP 105-20 standard [47].

### 3.5. Tensile Strength

The splitting tensile test was performed using four parallel Marshall specimens. The specimens were first conditioned in a water bath at 25 °C for 1.5 h. The test was then conducted at 25 °C using a loading machine with a constant rate of 50 mm/min [40]. The splitting tensile strength (*R_T_*) was calculated according to the standard formula:

(4) $$R_{T}=\frac{2P}{\pi td}$$

where *P* is the maximum load (N), *t* is the height of the specimen (mm), and *d* is the diameter of the specimen (mm).

### 3.6. Fatigue Cracking Performance

The uniaxial compression strength test was employed to examine the mixtures’ behavior under compressive loading. Cylindrical specimens with a diameter of 100 mm and a height of 100 mm were fabricated using a gyratory compactor [48]. The test was conducted at 15 °C with a loading rate of 2 mm/min. For the fatigue test, three stress levels (0.3, 0.4, and 0.6) were applied at a loading frequency of 10 Hz [45].

## 4. Results and Discussions

### 4.1. Rheological Properties

**Figure 3:**
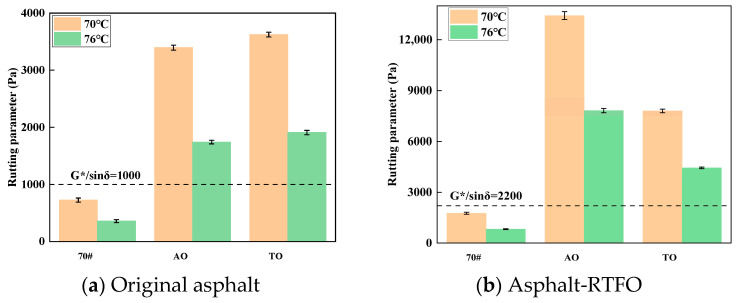
Temperature sweep of original asphalt (**a**) and asphalt after Rolling Thin Film Oven (RTFO) aging test (**b**).

The rutting parameter (G*/sinδ) is a critical indicator in the Superpave specification system for evaluating the high-temperature performance of asphalt binders. Physically, G*/sinδ represents the ratio of the complex shear modulus (G*) to the sine of the phase angle (δ), which characterizes the binder’s resistance to permanent deformation under repeated loading at high temperatures. A higher G*/sinδ value indicates better elastic response and enhanced resistance to rutting. Figure 3 shows the temperature sweep results of original asphalt and RTFO-aged asphalt.

Figure 3 illustrates that the rutting parameters (G*/sinδ) for the AO- and TO-modified binders at 70 °C are approximately 4.7 and 5.0 times higher than that of the 70# neat asphalt, respectively. Although the parameters for TO and AO decrease at 76 °C, their values remain superior to that of the base asphalt. Following RTFO aging, the rutting factor of the AO-modified binder increases significantly, whereas the increase for the TO-modified binder is relatively modest. The magnitude of change for AO remains pronounced at 76 °C, while that for TO is limited. Rubber particles can absorb the light components of asphalt and undergo swelling. The addition of light oil further promotes the swelling process. During this process, the rubber particles not only swell but also undergo a certain degree of degradation, with some rubber constituents dissolving into the asphalt, thereby effectively enhancing the high-temperature performance of the binder [49].

**Figure 4:**
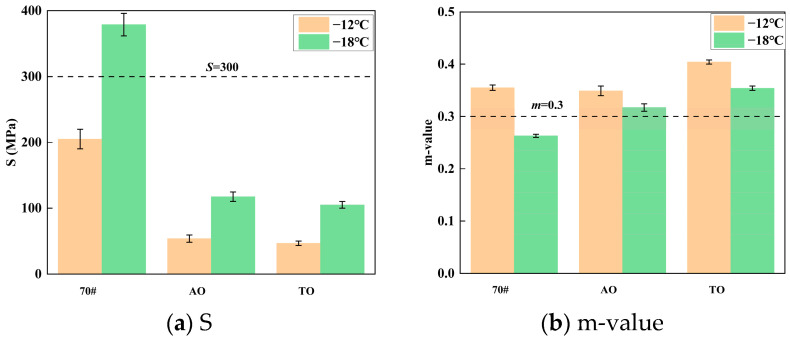
BBR test results of S (**a**) and m-value (**b**).

Creep stiffness (S) indicates the resistance of an asphalt binder to deformation under a constant load at low temperatures; a lower S-value corresponds to better low-temperature cracking resistance. The m-value represents the rate of stiffness change over time, where a higher value denotes greater stress relaxation capacity, thereby reducing thermal cracking potential. The BBR test results for the various asphalt binders at different temperatures are presented in Figure 4.

As shown in Figure 4, at a test temperature of −12 °C, the neat asphalt exhibits a creep stiffness of 231 MPa and an m-value of 0.375. However, its creep stiffness and m-value at −18 °C do not meet the standard specification requirements. In contrast, the tall oil and aromatic oil rubber composite-modified asphalts demonstrate creep stiffness values below 300 MPa and m-values exceeding 0.3 at −18 °C. Based on these creep stiffness results, the light oil/rubber-modified asphalts maintain favorable rheological properties at −18 °C. Both types of light oil/rubber composite-modified asphalts exhibit enhanced low-temperature performance compared to the neat asphalt. The light oil promotes the swelling of crumb rubber and improves its dispersion within the asphalt matrix.

### 4.2. Water Stability

**Figure 5:**
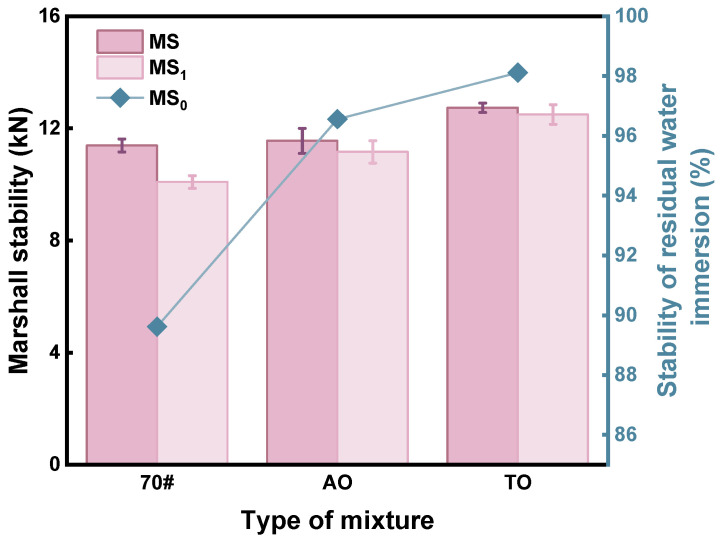
Immersion Marshall test result.

Moisture intrusion reduces the bond between asphalt and aggregates by penetrating poorly coated aggregate surfaces. When water reaches the asphalt–aggregate interface, it weakens the adhesive bond, leading to stripping of the binder. Under repeated vehicle loads on water-affected pavements, this moisture-induced damage can cause surface defects such as ruts, grooves, and potholes. The immersion test and the freeze–thaw splitting test are two methods used to evaluate the water stability of asphalt concrete properly and objectively. Figure 5 displays the Immersion Marshall test results.

The results are displayed in Figure 5. The strength of the AO conventional Marshall test is 11.56 kN, while that of TO is 12.74 kN. Both strengths are slightly higher than that of neat asphalt mixtures, with AO showing little difference overall and TO showing an increase of 11.2%. Compared to AO, TO intensity increases by 10.2%. Both modifiers ensure that the Marshall test strength meets the specifications.

Marshall specimens under water immersion conditions all exhibited varying degrees of stability loss. The residual stability of neat asphalt, AO, and TO is 89.61%, 96.56%, and 98.11%, respectively. Compared to neat asphalt, both modified asphalts have substantially higher residual stabilities. This fact indicates that adding these two oils can improve the mixture’s water damage resistance. The residual stability of TO is greater than that of AO, suggesting that the water stability of TO has a more significant improving impact than AO. Light oil rubber-modified asphalt has a high viscosity and good bonding between asphalt and aggregates. The specimen retained good adhesion between the aggregates and asphalt even after submerging.

**Figure 6:**
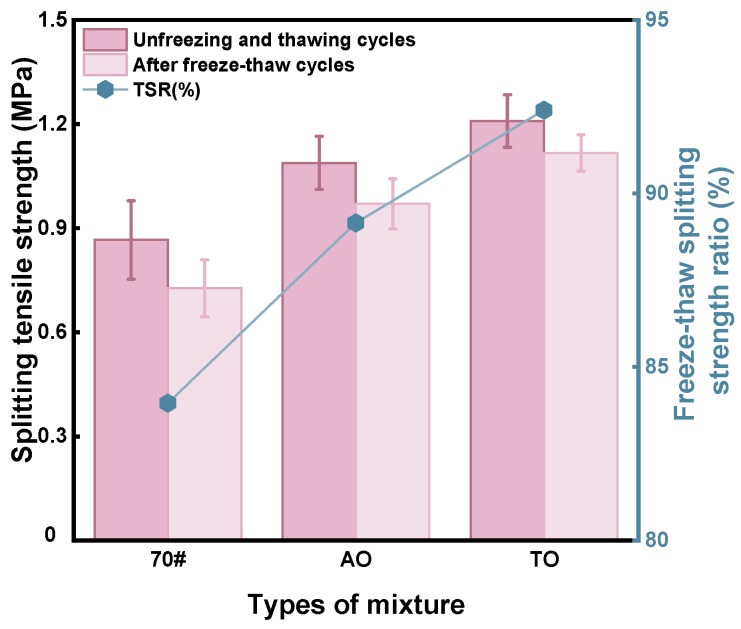
Freeze–thaw splitting test result.

The Freeze–thaw splitting test results are shown in Figure 6. After freeze–thaw cycles, all three asphalt mixtures show varying degrees of tensile strength reduction. Neat asphalt, AO, and TO have splitting strengths of 0.866 MPa, 1.088 MPa, and 1.209 MPa, respectively. The splitting strengths of neat asphalt, AO, and TO dropped to 0.727 MPa, 0.970 MPa, and 1.117 MPa, respectively, following the freeze–thaw cycle. Following freeze–thaw cycles, the splitting strength of TO is greater than that of neat asphalt. Additionally, the results show that the tensile strength ratio (TSR) of TO is 92.40% and the TSR of AO is 89.15%, both significantly higher than the neat asphalt’s 83.94%. Both modified asphalt mixtures meet the specified requirements (neat asphalt is 75%, modified asphalt is 85%). Both oil-modified asphalt mixtures have better split tensile strength and improved resistance to freeze–thaw damage, with the TO-modified asphalt mixture showing better water stability performance.

During the freeze phase, the expansion of water within the voids generates internal stress in the specimen. Unreacted rubber particles, distributed throughout the mixture due to their inherent elasticity and deformability, can absorb, store, and release deformation energy. This process helps mitigate the damage inflicted by freeze–thaw cycles on the mixture’s performance. Regarding the binder, the interactions between the crumb rubber and the light oil enhance the moisture resistance of the rubberized asphalt mixtures, leading to greater interfacial bonding strength between the asphalt and aggregates [39].

### 4.3. High-Temperature Stability

During summer, pavement surface temperatures can reach 50 °C to 70 °C under intense solar radiation, varying with geographic location, climate, and altitude. Rutting-induced thinning of the asphalt layer compromises the structural integrity of the surface course, accelerating subsequent distress mechanisms. This deterioration reduces road serviceability and poses safety risks. Therefore, wheel tracking tests were conducted in this study to evaluate the high-temperature stability of the asphalt mixtures, ensuring adequate resistance to permanent deformation.

**Figure 7:**
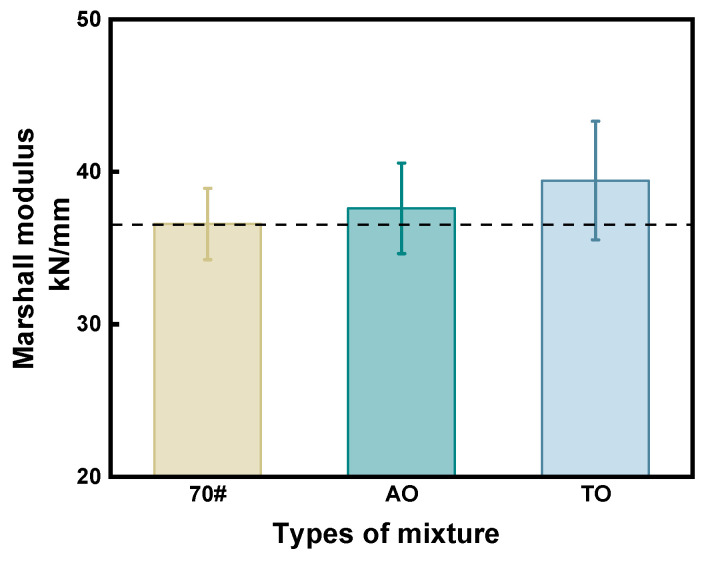
Marshall test results.

Marshall test results (Figure 7) show that the Marshall modulus of neat asphalt, AO, and TO are 36.8 kN/mm, 37.61 kN/mm, and 39.4 kN/mm, respectively. AO slightly increases by 2% compared to neat asphalt. TO exhibits a higher Marshall modulus than neat asphalt, showing a 7% increment. To some extent, the Marshall modulus reflects the high-temperature stability of asphalt mixtures. The inclusions of rubber and two different types of oils enhance the resilience of asphalt mixtures at high temperatures. Significant volatilization of AO during the asphalt preparation process causes a decrease in AO viscosity, impacting the mixture’s performance. The low level of adhesion between aggregates and asphalt causes a decline in performance at high temperatures.

The results obtained from the Marshall test cannot reflect the actual pavement service conditions. As a result, high-temperature rutting tests are used for high-temperature performance evaluation. Therefore, continuing to use high-temperature rutting tests is essential for evaluating the high-temperature performance of pavements.

**Figure 8:**
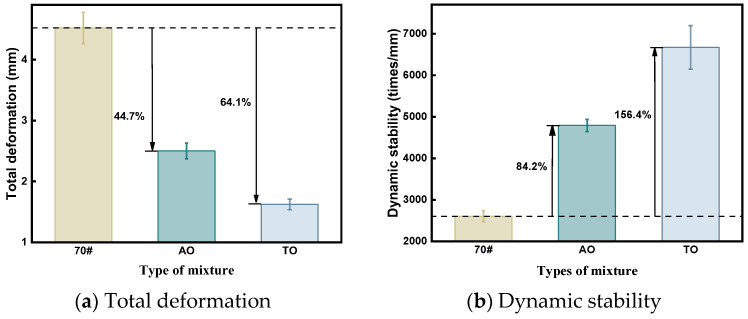
Rutting test result.

Figure 8 displays the rutting test results. The high-temperature performances of AO and TO asphalt mixtures compared to neat asphalt exhibit a significant improvement. Specifically, in the rutting test results, the total deformation values of 2.501 mm (related to AO) and 1.622 mm (associated with TO), representing reductions of 44.7% and 64.1%, respectively, compared to the 4.521 mm linked with neat asphalt. Regarding dynamic stability, AO is 4791 times/mm compared to the neat asphalt, which is an increase of 84.2%. TO increases by 156.4% to 6670 times/mm compared to the neat asphalt. The anti-deformation abilities of the two oil-modified asphalt mixtures under high-temperature conditions considerably improve. The improvement effect of TO-modified asphalt is better than AO-modified asphalt, similar to the analysis results in the Marshall test.

At high temperatures, asphalt loses a portion of its light components. The addition of light oil compensates for this loss by promoting the swelling and subsequent degradation of the crumb rubber, which replenishes the light component content within the binder matrix. Furthermore, under loading, the rubber particles within the aggregate structure undergo volumetric compression. This action absorbs energy, provides structural support, and mitigates damage to the specimen [39]. However, the volatility of aromatic oil weakens its viscosity-reducing effect and can lead to inferior aggregate adhesion [38,50]. Consequently, tall oil remains the optimal choice for this application.

### 4.4. Low-Temperature Cracking Test

Cold regions prioritize the low-temperature performance of asphalt pavement, with cracking being the most common low-temperature failure. As the temperature gradually decreases due to asphalt being a viscoelastic material, the internal temperature stress changes are complex. Due to the phenomenon of thermal expansion and contraction of materials, shrinkage occurs in rubber asphalt pavement, making it prone to cracking and other failures.

**Figure 9:**
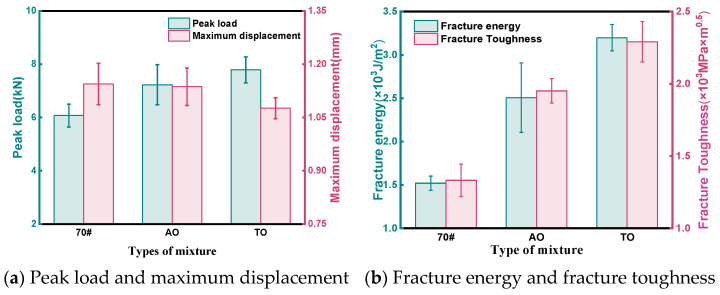
SCB test result.

The intuitive results of the experiment (Figure 9) show that AO and TO have smaller deformations under peak load. From a calculation perspective, the fracture energies of AO and TO compared to neat asphalt increase by 64.9% and 110.5%, respectively, while the fracture toughness values rise by 46.5% and 71.9%.

Rubber powder significantly enhances the fracture toughness of asphalt mixtures. The degree of improvement is more pronounced at lower test temperatures. This increase in fracture toughness reflects the specimen’s improved capacity to resist crack propagation. The enhancement is attributed to the unreacted rubber powder, which acts as a flexible aggregate, effectively replacing a portion of the fine aggregate fraction.

### 4.5. Tensile Strength

During the splitting test, the specimen’s intermediate stress state is more similar to the stress condition at the pavement’s base under load. Thus, the splitting test detects the mechanical properties of asphalt mixtures. The addition of rubber increases the viscosity of modified asphalt, resulting in a stronger bonding force of prepared asphalt mixtures. The shear temperature and shear rate during the preparation of modified asphalt do not reach the conditions for the complete reaction between rubber and asphalt. This phenomenon reduces the bonding performance of unreacted rubber particles in modified asphalt. The addition of two types of oil can enhance the splitting strength of modified asphalt by better encapsulating aggregates and rubber particles.

**Figure 10:**
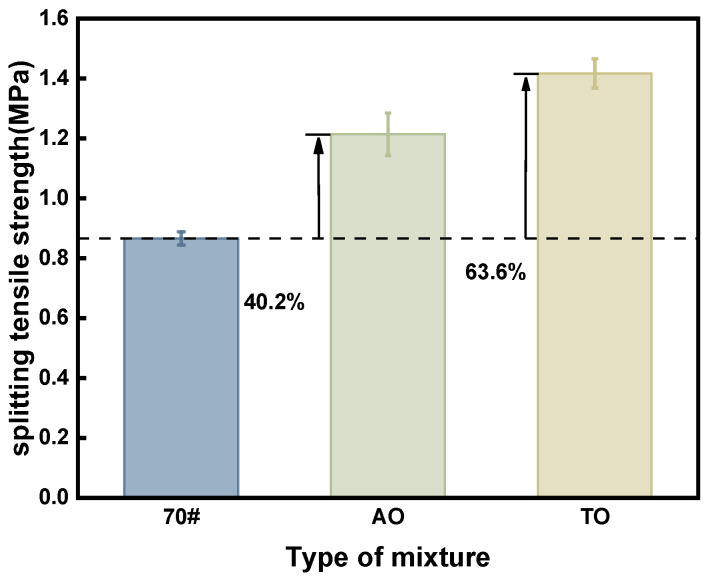
Splitting test result.

As illustrated in Figure 10, the tensile strengths of AO and TO are 1.213 MPa and 1.416 MPa, respectively. Both types of oil-modified rubber asphalt mixtures have higher splitting tensile strengths than neat asphalt, with increases of 40.2% and 63.6%, respectively, attributed to the combined action of rubber and light oil.

### 4.6. Fatigue Cracking Performance

When the repetitive loading fatigue stress induced by the load exceeds the strength of the pavement mixture material, it can lead to pavement damage. During the uniaxial compression process, asphalt mixture not only withstands the adhesive force of asphalt mastic and the resistance generated inside the aggregate but also experiences relative displacement and dislocation between mineral particles under pressure, resulting in inter-aggregate frictional resistance and interlocking forces. Consequently, this leads to the asphalt mixture having a greater compressive strength.

**Figure 11:**
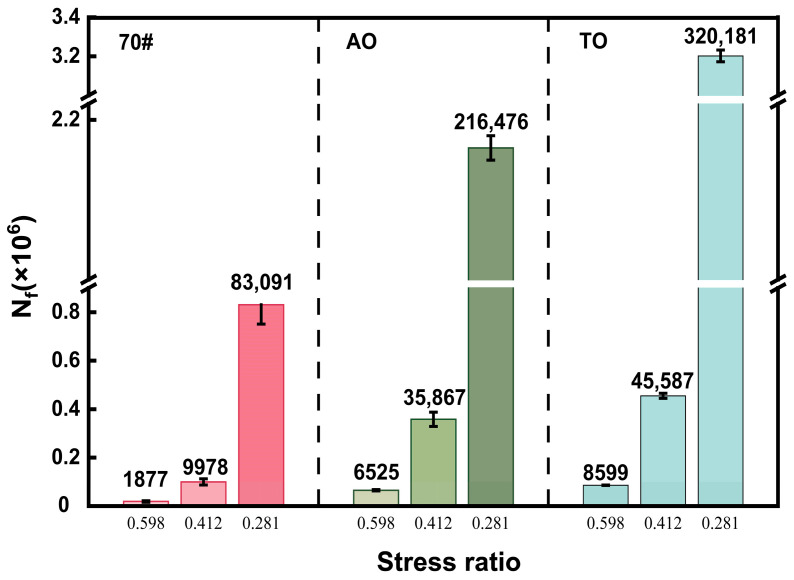
Uniaxial compression strength test result.

The fatigue life, N_f_ (in million cycles, ×10^6^), is plotted on the *y*-axis of Figure 11. The uniaxial compression fatigue test results indicate that under all three stress ratio conditions, the fatigue cycles for both the AO and TO exceed those of the 70# neat asphalt mixture, demonstrating their superior fatigue resistance. Furthermore, the TO exhibits a significantly longer fatigue life than the AO. At a stress ratio of 0.598, the fatigue life of the TO mixture is 8599 cycles, which is 31.8% higher than the 6525 cycles for the AO. At a stress ratio of 0.412, the fatigue life increases to 45,587 cycles for TO compared to 35,867 cycles for AO, an improvement of 27.1%. The most notable enhancement occurs at a stress ratio of 0.281, where the TO mixture’s fatigue life reaches 320,181 cycles, representing a 47.9% increase over the 216,476 cycles of the AO.

The enhanced fatigue performance can be attributed to the interaction between the crumb rubber and the light oil. Part of the rubber absorbs aromatic compounds from the oil, leading to swelling and promoting component migration. The rubber hydrocarbons and non-polar molecules (e.g., light components of the oil) intermix, while friction between polar molecules accelerates chemical reactions under heated conditions. Meanwhile, unreacted rubber particles act as elastic components within the matrix. These particles absorb stress during the loading phase of the fatigue test and release it during unloading. This repeated energy absorption and release mechanism significantly mitigates fatigue damage accumulation [51].

## 5. Conclusions

This study conducted a systematic experimental evaluation to compare the effects and enhancement mechanisms of two bio-based light oils—aromatic oil (AO) and tall oil (TO)—on the performance of crumb rubber-modified asphalt mixtures. Based on quantitative test data, the following main conclusions are drawn:

(1) High-Temperature Stability: Wheel tracking tests demonstrated significantly improved deformation resistance for the light oil-modified mixtures. The total deformation of AO and TO mixtures was 44.7% and 64.1% lower than that of the neat asphalt mixture, respectively, while their dynamic stability increased by 84.2% and 156.4%. Rheological tests revealed that the G*/sinδ values for AO and TO binders at 70 °C were 4.7 and 5.0 times that of the neat asphalt, confirming their superior rutting resistance at high temperatures.
(2) Low-Temperature Crack Resistance: SCB tests showed that the fracture energy of AO and TO mixtures increased by 64.9% and 110.5%, and fracture toughness improved by 46.5% and 71.9%, respectively. BBR test results indicated that at −18 °C, both modified binders had creep stiffness values below 300 MPa and m-values greater than 0.30, significantly outperforming the neat asphalt. This confirms that light oils promote rubber swelling and dispersion, effectively enhancing low-temperature cracking resistance.
(3) Moisture Susceptibility: In the immersion Marshall test, the residual stability values for AO and TO mixtures reached 96.56% and 98.11%, respectively. The freeze–thaw splitting strength ratios were 89.15% for AO and 92.40% for TO, both meeting specification requirements and significantly exceeding the performance of the neat asphalt mixture. This improvement is attributed to the increased binder viscosity and enhanced aggregate coating provided by the light oils, coupled with the elastic deformation of crumb rubber particles mitigating frost damage.
(4) Mechanical and Fatigue Performance: Splitting tests indicated tensile strength improvements of 40.2% for AO and 63.6% for TO mixtures. At a low stress ratio of 0.281, the fatigue life of AO and TO mixtures was 2.6 and 3.9 times that of the neat asphalt mixture, respectively, demonstrating superior durability. This enhancement originates from the crosslinked network structure formed by the rubber–light oil–asphalt interaction and the stress-buffering effect of unreacted rubber particles during load cycles.
(5) Overall Comparison: While both light oils significantly improved mixture performance, tall oil (TO) demonstrated superior effectiveness. This is primarily because the volatility of aromatic oil during preparation may weaken bonding, whereas tall oil offers better chemical stability, resulting in higher interfacial bond strength. The proposed formulation of 5% light oil with 20% crumb rubber achieved comprehensive performance enhancement while allowing for reduced mixing temperatures. This provides an efficient pathway for recycling waste tires and aligns with the development needs of green, long-life pavements. Tall oil is recommended as the preferred light oil type for rubber-modified asphalt.

This study focused solely on aromatic oil and tall oil; the influence of light oils from other sources was not explored. Future work will prioritize investigating the effects of aggregate gradation and crumb rubber content on mixture performance.

## Figures and Tables

**Table 1:** Fundamental characteristics of 70# asphalt.

Properties	Measured Value	Reference Standard
Penetration at 25 °C (0.1 mm)	67.4	AASHTO T 49 [30]
Softening point (°C)	48.5	AASHTO T 53 [31]
Ductility at 10 °C (mm)	31.4	AASHTO T 51 [32]
Viscosity at 135 °C (mPa·s)	462.3	AASHTO T 316 [33]
G*/sinδ of RTFO aged asphalt (64 °C)	2483.3	AASHTO T 315 [34]

**Table 2:** Detailed parameters of aromatic oil.

Properties	Unit	Results
Density (15 °C)	g/mL	1.01
Dynamic viscosity (100 °C)	mm/s^2^	24.6
Aromatic content	%	95
Water content	%	35
Moisture content	%	0.03

**Table 3:** Detailed parameters of tall oil.

Properties	Unit	Results
Density (15 °C)	g/mL	0.96
Acid value	mg KOH/g	191.8
Rosin acid content	%	29.45
Fatty acid content	%	69.33
Saponification value	mg KOH/g	184.6

**Table 4:** Detailed parameters of rubber.

Properties	Requirements	Results
Density (g/cm^3^)	≤1.2	1.2
Water content (%)	≤1.0	0.5
Metal content (%)	≤0.05	0.02
Ash (%)	≤10	7
Acetone extract (%)	≤10	7
Rubber hydrocarbon content (%)	≥48	56
Carbon black content (%)	≥26	30

**Table 5:** Coarse and fine aggregate performances.

Items	Pilot Project	Result	Technical Requirements
Coarse aggregate	Crushed aggregate value (%)	12.5	≤18
	Los Angeles abrasion loss (%)	8.0	≤22
	Apparent specific gravity (g/cm^3^)	2.927	≥2.6
	Water absorption (%)	0.64	≤1.0
	Firmness	8.0	≤12
Fine aggregate	Apparent relative density	2.728	≥2.5
	Sand equivalent (%)	67	≥60
	Methylene blue value	1.3	≤2.5
	Angularity (s)	33.2	≥30

**Table 6:** Mineral powder performances.

Pilot Project	Results	Technical Requirements
Apparent relative density (g/cm^3^)	2.68	≥2.5
Appearance	no clumps	no clumps
Water absorption (%)	0.54	≤1.0

**Table 7:** Detailed information on the samples.

Crumb Rubber Content	Light Oil Content	Abbreviation
0%	0%	70#
20%	Aromatic oil (5%)	AO
20%	Tall oil (5%)	TO

**Table 8:** Marshall test results of three types of asphalt mixtures.

Type of Mixture	Asphalt–Aggregate Ratio (%)	Air Voids (%)	VMA (%)	VFA (%)	Stability (kN)	Flow Value (mm)
70#	4.0	3.164	10.616	66.118	9.753	3.006
	4.5	2.695	10.472	77.363	12.800	3.305
	5.0	1.648	10.749	84.682	10.950	3.568
AO	5.7	4.368	14.363	69.590	10.185	2.862
	6.0	3.653	14.199	74.297	12.125	2.256
	6.3	3.531	14.724	76.039	10.343	2.962
TO	5.7	4.205	14.890	71.770	9.067	2.899
	6.0	3.660	14.741	75.199	12.750	2.531
	6.3	3.424	15.092	77.317	10.710	2.667

## Data Availability

The original contributions presented in this study are included in the article. Further inquiries can be directed to the corresponding authors.
